# Electrochemical Aptasensors for Food and Environmental Safeguarding: A Review

**DOI:** 10.3390/bios8020028

**Published:** 2018-03-23

**Authors:** Geetesh Kumar Mishra, Vinay Sharma, Rupesh K. Mishra

**Affiliations:** 1 Sabanci University Nanotechnology Research and Application Center, Orta Mahalle, 34956 Tuzla, Istanbul, Turkey; 2 Department of Biosciences and Biotechnology, Banasthali University, Rajasthan 304022, India; vinaysharma30@yahoo.co.uk; 3 Department of Nano-Engineering, University of California San Diego, La Jolla, CA 92093, USA

**Keywords:** aptamers, food safety, electrochemical sensors, environmental safeguard, aptasensors

## Abstract

Food and environmental monitoring is one of the most important aspects of dealing with recent threats to human well-being and ecosystems. In this framework, electrochemical aptamer-based sensors are resilient due to their ability to resolve food and environmental contamination. An aptamer-based sensor is a compact analytical device combining an aptamer as the bio-sensing element integrated on the transducer surface. Aptamers display many advantages as biorecognition elements in sensor development when compared to affinity-based (antibodies) sensors. Aptasensors are small, chemically unchanging, and inexpensive. Moreover, they offer extraordinary elasticity and expediency in the design of their assemblies, which has led to innovative sensors that show tremendous sensitivity and selectivity. This review will emphasize recent food and environmental safeguarding using aptasensors; there are good prospects for their performance as a supplement to classical techniques.

## 1. Introduction

The ever-increasing occurrence of contaminants in food and the environment is causing severe chronic illness and diseases. These circumstances point to the necessity of a rapid and multi-analyte analytical system capable of analyzing complex food and environmental samples. Most of the current analytical tools, which are being used for the analysis of food and environmental samples, still rely on antibody-based bio-molecular recognition events with target analytes. These assays were very well-known and have been established to attain the desired sensitivity and selectivity. However, the use of antibodies in multi-analyte detection techniques could encounter some limitations in the analysis of complex environmental and food samples due to the nature and synthesis of these protein receptors. In order to avoid some of these disadvantages, other recognition elements are being envisaged as alternatives to antibodies [1].

The understanding of nucleic acids, particularly RNA, has directed us to the idea of selecting new nucleic acid ligands called aptamers. These aptamers are single-stranded DNA or RNA ligands that can be selected for different targets from a random pool of molecular sequences. These aptamers can be easily synthesized and labeled without affecting the affinity for their ligands and are more stable than antibodies, which is a well-desired attribute for any analytical technique in commercial applications. The process of selecting a particular molecular sequence for the synthesis of aptamers is known as the systematic evolution of ligands by exponential enrichment (SELEX). This process engages numerous cycles of selection and amplification of an oligonucleotide from a large library with different sequences. Upon incubation with specific target molecules and partitioning of the binding from non-binding molecules, selected oligonucleotides are amplified to create a new mixture comprising nucleic acid molecules with higher affinity towards the target [2,3,4].

Improvement of existing analytical procedures is important for ensuring rapid and sensitive detection of toxic compounds present in food and environmental samples. From this perspective, biosensors have been thoroughly explored as potential analytical tools for providing fast response, sensitive detection, and anticipated specificity towards the analyte of interest, lower cost of sample analysis, and ease of portability as compared to commonly used classical analytical methods such as chromatography, ELISA, PCR, or microbial culture for bacterial contamination. The electrochemical aptasensors have emerged as a promising tool among the other reported biosensors, owing to their sensitivity and specificity for the recognition of target analytes. These electrochemical aptasensors are further advanced by using labeled redox probes, which involves an aptamer-containing redox molecule coupled with the electrode surface [5]. In these types of aptasensors, upon interaction with target analytes, conformational changes occur to the immobilized aptamer, which directs the change in the competence for electron transfer resistance between the electrode surface and electroactive solution [6]. Similarly, the concentration of target analytes is recorded by measuring the faradic current generated upon conformational changes in aptamer structures [2]. Aptamers have been widely explored for the detection of various molecules including organic dyes, amino acids, antibiotics, peptides, proteins, vitamins, and also whole cells or micro-organisms such as bacteria [7].

In recent years several detailed studies have reported on the development of aptamer-based biosensors or the combination of aptamers with different nanomaterials for biosensing applications in food and environmental analysis [8,9,10,11,12]. In this review, we will focus on recently reported electrochemical aptasensors applied to assessing various food and environmental contaminants.

## 2. Electrochemical Detection Principles

In most electrochemical biosensing measurements, extractions of electrical properties for biological entities are electrochemical in nature, where a bioelectrochemical component serves as the transduction element. Generally in electrochemical biosensors, investigated reactions would either generate a measurable current, charge accumulation, or conductance, which could be measured by amperometric, potentiometric, or conductometric techniques, respectively. Biological or chemical reactions are generally detected on a close proximity of electrode surface. Based on the electrochemical properties of a specific electrode surface, detection techniques are chosen. Electrochemical techniques require a reference, auxiliary, and a working electrode or sensing electrode. The reference electrodes are mostly made from silver chloride and fixed at a distance from the reaction site to maintain a stable potential. The sensing electrode acts as a transduction element in the biochemical reaction, whereas a counter electrode creates contact between the electrolytic solution and electrode surface to apply current to the sensing electrode.

### Design of an Electrochemical Aptasensor

The design of a general electrochemical biosensor begins with the sensor surface, which needs to be conductive and transparent if the detection technique is to be seen visually. A surface-covering layer has to provide hydrophobicity, inertness, and functional groups for immobilization of the biorecognition elements, which may be an aptamer or antibody depending on the type of electrochemical biosensor. Biorecognition elements are immobilized on the sensor surface to specifically capture target molecules that need to be detected. Many electrochemical techniques employ labeled biorecognition elements to enumerate the binding events. In most of these cases, the label is an enzyme that catalyzes binding reactions when the immobilized biorecognition element is not able to convert the charge transfer process to a measurable signal. In some reactions, mediators are being used in the form of redox probes, if the reaction takes place away from the electrode interface, it can transfer electrons between the reaction site and the surface.

Construction of an electrochemical aptasensor begins with immobilization of an aptamer on a conducting substrate. Aptamers have been immobilized on these electrodes, preferably gold (Au) and carbon-based electrodes, either by direct chemisorption or through the functionalization and surface modification of the electrode surface by chemical cross-linking. Typically, the electrode surfaces were first functionalized to permit the attachment of a suitable amount of aptamer and improve the electron-transfer properties, or immobilization of electroactive probes that can be used as correspondents during the binding events. Immobilization of aptamers on the transducer surface is an influential step for determining the performance of developing aptasensor. Several approaches were described for ensuring adequate stability, surface coverage, and maintaining the binding affinity of the aptamer, including chemisorption of thiolated aptamers on Au electrodes; attachment of biotinylated aptamer to avidin-modified sensor surfaces; immobilization of an azide-ended aptamer to alkyne-modified surfaces; covalent immobilization of amine-ended aptamers by amine coupling to carboxyl groups in functionalized surfaces; covalent immobilization of amine ended aptamer to functionalized surfaces containing amine groups using glutaraldehyde; etc. [13,14].

A diverse range of surface functionalization strategies were explored, ranging from simple electrochemical deposition of diazonium salts and Au nanoparticles (AuNPs) on carbon-based electrodes, to modification with conducting polymers and the sequential deposition of several nanocomposites. Nanomaterials and nanocomposites have been used to increase the electroactive area, increase the aptamer loading on the electrode surface, and provide a three-dimensional support that facilitates aptamer immobilization and minimizes steric hindrance [8]. Among the various electrochemical methods, electrochemical impedance spectroscopy (EIS), voltammetry (which includes cyclic voltammetry (CV), differential pulse voltammetry (DPV), square wave voltammetry (SWV), and linear sweep voltammetry (LSV)), field effect transistor (FET), and potentiometry have been used for aptasensor development [15,16].

**Figure 1:**
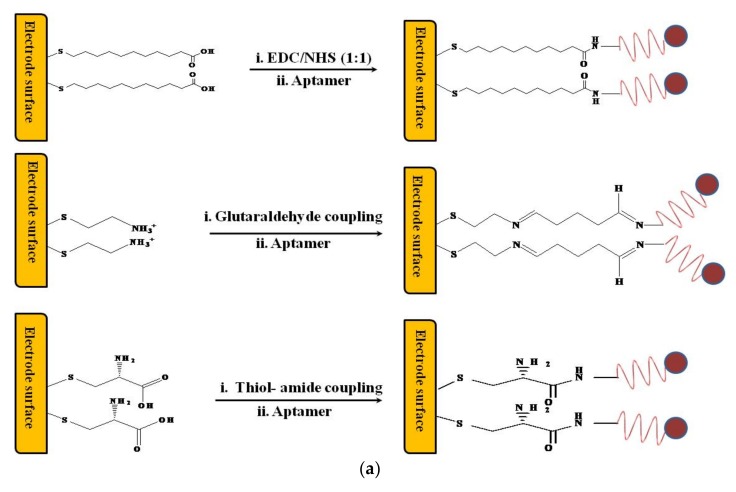

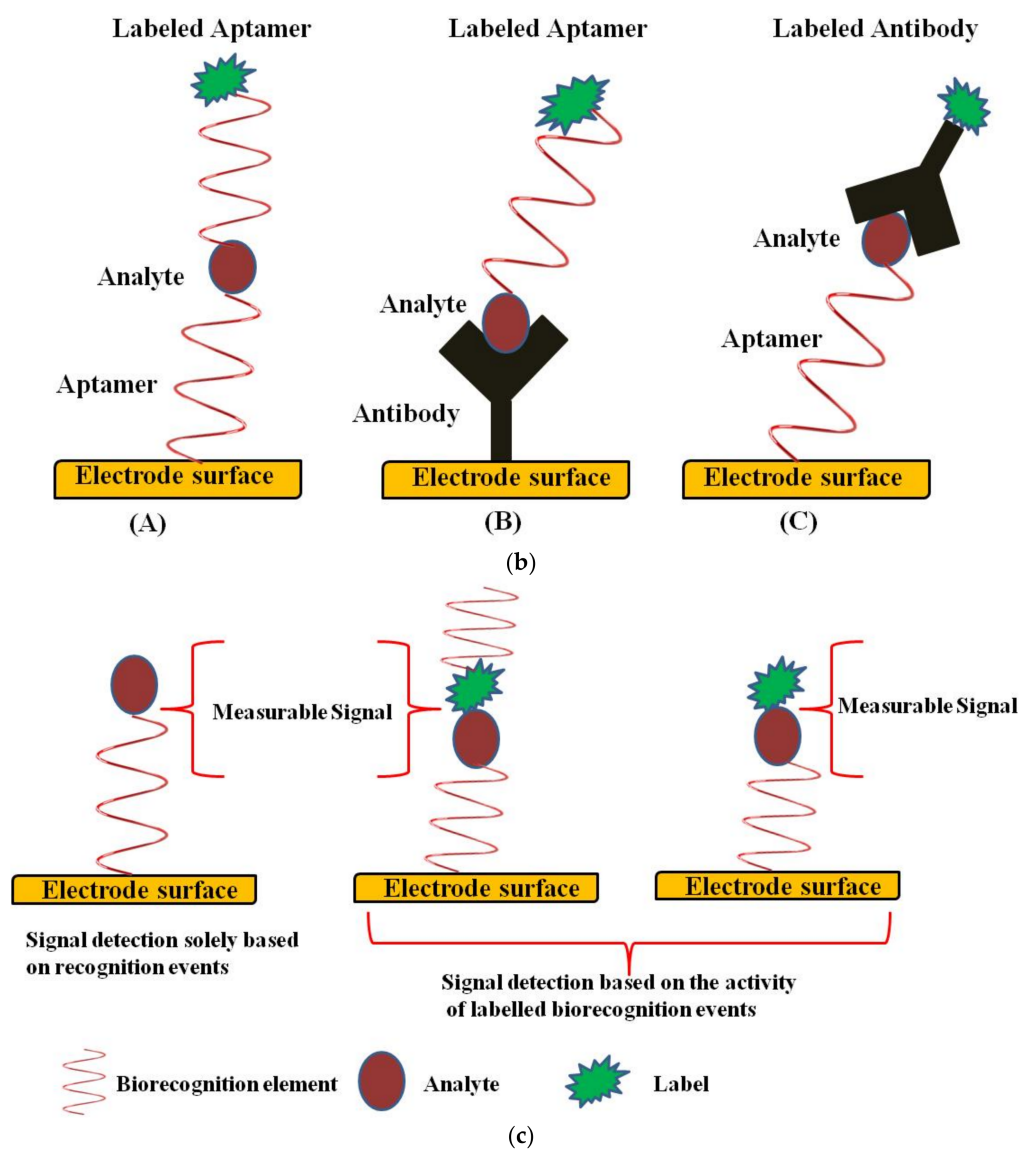
(**a**) Schematic representation of various immobilization strategies for electrochemical aptasensor construction. (**b**) Schematic representation of various sandwich-type immobilization strategies for electrochemical aptasensor construction: (**A**) aptamer-analyte-labeled aptamer sandwich; (**B**) antibody-analyte-labeled aptamer sandwich; (**C**) aptamer-analyte- labeled antibody sandwich. (**c**) Schematic representation of label-free and labeled detection mechanism using aptamers.

Likewise, varied immobilization and labeling methods have been applied to the construction of electrochemical aptasensors. These methods can be covalent coupling of aptamers to themselves or to complementary sequences, indirect attachment of aptamers, or coupling of aptamers and antibody mixtures to the electrode surface. Depending on the forms of electrochemical assay, it can be either a signal-on or a signal-off technique. In some cases, redox probes are also being used as tagged entities to the aptamers. Related schemes for various immobilization/labeling strategies in Figure 1a, various sandwich-type immobilization strategies in Figure 1b, and detection mechanisms based on labeled and label-free aptamers have been presented for electrochemical aptasensor construction in Figure 1c.

## 3. Electrochemical Aptasensors for Food Safeguarding

Electrochemical aptasensors have been thoroughly explored as a potential analytical platform providing the desired portability, rapidity, sensitivity, and selectivity in food samples analysis. Most importantly, they confer ease of operation as compared to the conventional analytical methods. Abundant aptasensors have been reported for the detection of microbes, food allergens, mycotoxins, marine toxins, antibiotics, and pesticides. Newly reported methods account for the increased sensitivity of the detection method by exploring a variety of nanomaterials and engineered aptamers for food safety and control. Testimonies for some of the recently reported aptasensors have been described below for food safeguarding purposes.

### 3.1. Electrochemical Aptasensors for Microorganisms

Microorganisms, such as bacteria, viruses, and parasites, might contaminate food and cause severe public health problems. Recently, numerous aptasensors have been reported using important tools including impedimetric, potentiometric, and voltammetric techniques for the detection of several bacteria and parasites. The accomplishment of sensitive detection limits for microbes was assisted by integrating nanomaterials like grapheme oxide (GO), carbon nanotubes (CNTs), and gold nanoparticles (AuNPs) or by amplification of enzyme-labeled probes [5]. The detection of the most common food pathogen, *Salmonella typhimurium*, was recently reported in pork, exploring AuNPs and GO using EIS-based technique with a limit of detection (LOD) of 3 CFU/mL [17]. In another work, the authors reported the detection of *Staphylococcus aureus* in pig skin using single-walled carbon nanotubes (SWCNT) by applying the potentiometric technique and achieved 800 CFU/mL [18]. Besides these detection techniques, some recently reported methods have explored various nanomaterials for food sample analysis. Recently, Izadi and co-workers demonstrated an electrochemical DNA-based biosensor for *Bacillus cereus* in milk and infant formula. They used AuNPs to prepare a modified pencil graphite electrode that could detect *Bacillus cereus* as low as 10^0^ CFU/mL [19]. In another research work, a DNA biosensor for the detection of pathogenic bacteria *Aeromonas hydrophila* in fish and vegetables was developed based on the electrochemical technique [20]. An impedimetric biosensor was demonstrated for the rapid detection of *Salmonella typhimurium* based on the poly [pyrrole-co-3-carboxyl-pyrrole] copolymer-supported aptamer in a spiked food sample [21]. The aptamer was conjugated with Ag nanoparticles for the detection of *Staphylococcus aureus* using a dual aptamer-based sandwich electrochemical sensor [22]. Future strategies for detection of these microbes in food samples might apply a combination of various aptamers rather than single aptamers for better sensitivity.

### 3.2. Electrochemical Aptasensors for Food Allergens

Food allergy is an undesirable immune reaction towards some food ingredients. Most reported allergens are proteins that might be significantly modified during food processing. The presence of various food allergens and their interaction with food elements is a challenge in the development of sensitive analytical methods for the detection of these allergens in complex food matrices [10,23]. Ever-increasing awareness of food safety and regulations has led to the development of a variety of modern analytical technologies. Until now, various detection methods have been developed using aptamers and electrochemical techniques. Lysozymes have been studied extensively as the most common food allergen since they are used as antimicrobial and fining agents in the food industry. Lysozymes are also used in beer and sausage production to control the growth of bacterial species. One such demonstration of lysozyme detection was recently reported in wine using an electrochemical aptamer–antibody sandwich assay [24]. Although the reported method has an intricate process of analysis, it was able to detect lysozymes as low as 4.3 fM in spiked wine samples, with good stability and reproducibility.

### 3.3. Electrochemical Aptasensors for Fungal Toxins

Fungal toxins, also known as mycotoxins, are chemically heterogeneous toxic secondary metabolites of fungal origin with a broad range of toxic effects. Several mycotoxins have been studied in the recent past; among them, aflatoxins and Ochratoxin A (OTA) have been studied extensively. Detection of mycotoxins using aptamer-based tactics has been an active research area in designing electrochemical techniques. Among the various mycotoxins, OTA, aflatoxin M1 (AFM1), and aflatoxin B1 (AFB1) have been the most extensively studied analytes for the detection of electrochemical aptasensors. A simple and sensitive aptasensor was recently developed for the detection of OTA using covalent immobilization of OTA aptamers on screen-printed electrodes (SPE). The authors reported a linear detection range for OTA between 0.15 and 2.5 ng/mL using an impedimetric technique [25]. In an additional impedimetric aptasensor-based approach, the authors reported the detection of AFM1 in raw milk using covalent immobilization of a hexa-ethylene glycol-modified aptamer on SPE. They reported a reproducible detection range between 2 and 150 ng/L. Furthermore, the authors analyzed spiked milk samples for AFM1 and the obtained results correlated well with other established commercial methods [26]. More recently, an electrochemical aptasensor using a methylene blue (MB)-labeled probe was reported for the detection of AFB1. The authors explored functional graphene oxide as a signal enlarging platform cast on a screen-printed carbon electrode (SPCE). In this method, an MB-tagged aptamer was covalently immobilized on a SPCE via hexamethylenediamine (HMDA) and carbodiimide (amide) bonding. Analyte detection was realized by an aptamer-conjugated redox probe, which goes through a confirmation structural change upon binding with AFB1. The method could reach AFB1 detection as low as 0.05 ng/mL with excellent linearity between 0.05 and 6.0 ng/mL in alcoholic beverages using the DPV technique. This method could be helpful for the rapid and sensitive detection of AFB1 and other mycotoxins in alcoholic beverages [6]. Catanante et al. developed a folding mechanism-based electrochemical aptasensor for OTA using MB-tagged anti-OTA aptamers. In this work, they demonstrated various aptamer coupling strategies using HMDA, polyethylene glycol, and diazonium coupling. The best performance was recorded through oxidation of amines using HMDA on SPCE with LOD of 0.01 ng/mL [27]. The OTA detection was also done efficaciously in cocoa beans by Mishra et al. using a DPV-based aptasensor [28]. In this work, a molecular-imprinted polymer was used to filter the cocoa samples and then detected electrochemically by aptasensors.

### 3.4. Electrochemical Aptasensors for Antibiotics

The increasing use of antibiotics in combination with other drugs for the treatment of inflammatory diseases or as a feed additive has led to antibiotic resistance of pathogenic bacteria and antibiotic accumulation in food elements. Monitoring of antibiotic residues in food is very important for human consumption; even the minute presence of these residues can trigger an allergic reaction in hypersensitive individuals [29]. The increasing occurrence of antibiotic contamination in animal-derived foods, i.e., milk, meat, etc., has led to significant attention to and development of early and specific methods for detecting antibiotic residues using aptamer-based electrochemical sensors. Several antibiotic groups such as aminoglycosides and tetracycline are being used as broad-spectrum antibiotics in the food industry to prevent bacterial contamination and fouling of food. Recently, a disposable and portable impedimetric aptasensor was reported for the detection of kanamycin residue in milk samples. The target detection was based on specific recognition by a kanamycin-aptamer covalently immobilized on SPE. The authors reported a good dynamic range (1.2–600 ng/mL) for kanamycin, with linearity between 1.2 and 75 ng/mL. The LOD of the developed aptasensor was determined to be 0.11 ng/mL in milk samples [30]. Similarly, Zhou and co-workers developed a label-free electrochemical aptasensor for antibiotic kanamycin residue determination in milk. The SWV was used to generate a current response that was further utilized to conclude the concentration of kanamycin. The working range of the aptasensor was established to be 10–2000 nM. The biosensing approach of the aptasensor was based on the conformational change of a kanamycin-specific aptamer self-assembled on the gold electrode surface [31]. In alternative work for kanamycin sensing, a signal amplification approach was adopted based on thionine-functionalized graphene and graded nanoporous PtCu, and the detection limit could go down to 0.42 pg/mL [32]. Plentiful aptasensors were developed for other antibiotic residues such as an aptasensor for tetracycline detection developed using alginate film/reduced graphene oxide and magnetite particles coupled to a carbon electrode. This aptasensor was found to be stable for up to two weeks, with 95.86% of its initial activity [33]. An innovative aptasensor for recognition of chloramphenicol in honey was established based on a target-induced strand release (TISR). CAP aptamer was immobilized on electrode and then hybridized with the complementary biotinylated detection probe to form an aptamer/DNA duplex. In the presence of CAP, the TISR resulted in the dissociation of a biotinylated detection probe from the electrode. The literature showed that various antibiotic residues have been tested using several new protocols exploiting novel nanomaterials and novel surface modification protocols on the electrode surface. In one approach, Prussian a blue-chitosan-glutaraldehyde-modified electrochemical aptasensor for the detection of tetracycline was developed to achieve a label-free signal. The Prussian blue-chitosan-glutaraldehyde system acted as the signal gauge to improve the sensitivity of sensor which was lower down to 3.2 × 10^−10^ M [34]. Nanomaterial multi-walled carbon nanotubes (MWCNT) were integrated to develop another electrochemical aptasensor for the detection of tetracycline. This nanotube was dropped on the glassy carbon electrode to couple aptamer and to construct the aptasensor with LOD of 5 × 10^−9^ M [35].

The majority of the reported aptasensors have exhibited high sensitivity and a suitable linear range for quantitative analysis of antibiotic residues and were verified to be reproducible and fit for sample analysis. A summary of the reported electrochemical aptasensor applied for food safeguarding is presented in Table 1.

## 4. Electrochemical Aptasensors for Environmental Safeguarding

Recent years have witnessed a growing need to examine environmental pollutants and keep a constant watch on them. Water and air are the main commodities getting regularly contaminated, which may have a severe effect on human and animal life. These pollutants may have gentle to harsh effects, which may be visible immediately or only after a long time. Some of the pollutants have lethal effects and could lead to a public health crisis [36]. Environmental pollutants, which should be under constant monitoring, can be generally categorized as organic and inorganic pollutants, which include heavy metals, pesticides, pharmaceutical products, and toxins of biological origin. Industrialization and a growing population are the main causes of environmental pollution, which poses a risk to human life and the ecosystem. It is therefore important to develop early detection methods as well as novel analytical tools to identify and quantify these environmental contaminants, chemical or biological toxins, and their chemical compositions at very low levels with low cost and high efficiency. Progress in the field of electrochemical biosensors is intended to develop sensitive and robust methodologies that are easy to operate, economical, and rapid for sample analysis. Such progress offers better analytical techniques with a reduced environmental impact. The discovery of aptamers offered breakthrough opportunities in biosensor development. Aptamer-based electrochemical biosensors can replace conventional methods for the detection and estimation of environmental concerns [37,38]. Recently developed electrochemical aptasensors for the monitoring of environmental samples are discussed in the sections below.

### 4.1. Electrochemical Aptasensors for Heavy Metals

Accumulation of heavy metals in the environment and food chain is increasing above safe levels due to rapid industrialization, modern agricultural activities, mining, and sludge dumping, which pose a severe threat to human and animal health. Therefore, detection and screening of heavy metals is gaining more consideration these days. Heavy metals such as arsenic (As), lead (Pb), and mercury (Hg) can cause severe toxic effects, including heart disease, skin lesions, and damage to the endocrine and nervous system, upon sustained exposure. Extensive research is in progress for the development of highly sensitive and selective biosensors exploring aptamers for the detection of these heavy metals at very low levels. So far, numerous electrochemical aptasensors have been developed to detect heavy metals for environmental applications and are projected to control the permissible levels. Recently, a simple and reusable electrochemical aptasensor was reported for sensitive and selective detection of mercury ions (Hg^2+^). The aptasensor was based on thymine-Hg^2+^-thymine (T-Hg^2+^-T) coordination chemistry and nanoporous gold (NPG) for signal amplification and could detect Hg^2+^ in the range of 0.01–5000 nM, with an LOD of 0.0036 nM. The reported limits of the aptasensor were much lower than the maximum contamination level for Hg^2+^ in drinking water defined by the regulatory agencies. Furthermore, the reported electrochemical aptasensor could be easily regenerated upon the addition of cysteine and Mg^2+^. The aptasensor was applied to detect Hg^2+^ from water and showed a promising prospective for on-site monitoring of Hg^2+^ in drinking water [39]. In another recently reported method, an ultra-sensitive and highly selective electrochemical label-free aptasensor was proposed for the quantitative detection of Hg^2 +^ in zeptomolar (zM) levels. The aptasensor was based on hybridization/dehybridization of double-stranded DNA (dsDNA) on a gold electrode. For the development of an aptasensor, the DPV technique was explored with Fe(CN)_6_^3−/4−^ as a redox probe. The electrochemical aptasensor was able to quantify Hg^2+^ ions in concentrations from 5 zM to 55 pM with an LOD of 0.6 zM. The reported LOD was compared to be close at single atom detection. The authors selectively detected Hg^2+^ in samples containing environmentally relevant metal ions without a complex process or costly resources [40]. A highly sensitive label-free detection of Hg^2+^ was illustrated based on the electrochemical aptamer detection method, which involved hybridization and dehybridization of ds-DNA using gold electrodes. A thymine-Hg^2+^-thymine complex is the basis for the development of this assay, which could quantify Hg^2+^ ions in concentrations up to 0.6 zM [40]. In another heavy metal aptasensor study, a label-free, signal-on sensor was developed for the detection of arsenite with an LOD of 0.15 nM [41]. An enzyme- and label-free method for lower-level detection of lead ions (Pb^2+^) was developed using an electrochemical aptasensor and a metal–organic framework, which was loaded with Ag–Pt particles that acted as electrocatalytic enhancers and achieved LOD as low as 0.032 pM with good stability [42].

In another approach, a novel strategy for selective and sensitive detection of Pb^2+^ was proposed using the amperometric technique. The aptasensor was based on a target-induced conformational switch. The ferrocene-labeled thiolated aptamer was self-assembled through gold-thiol bonding on the electrode surface, using mercaptoethanol blocking. The constructed aptasensor showed a linear response over the range of 5.0 × 10^−10^ M to 1.0 × 10^−7^ M with a LOD of 1.2 × 10^−10^ M to the log of Pb^2+^ concentration. Moreover, this approach has excellent selectivity for Pb^2+^ against other metal ions. The developed aptasensor showed good potential for Pb^2+^ detection in real herb samples [43].

### 4.2. Electrochemical Aptasensors for Pesticide Detection

Among the known pesticide groups, organophosphates (OPs) have attracted the most interest due to their highly toxic nature and their application as pesticides and chemical weapons. Various researchers have developed biosensors for the detection of toxic analytes such as paraoxon, parathion, and chlorpyriphos, exploiting various biomolecules such as enzymes, antibodies, and whole cells. Owing to their unique advantages of chemical recognition and ease of modification, aptamers have been extensively used in pesticide analysis. Electrochemical aptasensors have been developed to detect diverse targets by combining a variety of electrochemical techniques with aptamer-based signal conversion strategies. However, there are insufficient reports of electrochemical aptasensor for pesticide detection. Recently, a novel and ultrasensitive aptasensor was developed for the quantitative detection of chlorpyriphos. The sensitivity of the aptasensor was improved by using mesoporous carbon (OMC) functionalized by chitosan (OMC-CS) and ferrocene hybrid chitosan (CS)-dispersed MWCNTs-CS on the electrode surface. Under optimal conditions, the aptasensor was able to detect a wide linear range from 1 to 10^5^ ng/mL with a LOD of 0.33 ng/mL for chlorpyriphos. Reported chlorpyriphos aptasensors exhibited stable performance, with high selectivity and reproducibility in vegetables and fruits [44]. Likewise, several aptasensors have been reported for the detection of various other pesticides. Wang et al. designed a DNA aptamer that was able to detect up to four highly poisonous organophosphate pesticides, phorate, profenofos, isocarbophos, and omethoateas [45]. Fan et al. developed an aptasensor for sensitive and selective detection of acetamiprid based on electrochemical impedance spectroscopy. To improve the sensitivity of the developed aptasensor, the authors electrodeposited gold nanoparticles using cyclic voltammetry on the bare surface of the gold electrode. The developed aptasensor was successfully evaluated in real-life applications by determining acetamiprid in wastewater and tomatoes [46]. Although aptamers have been selected against a variety of pesticides, their possible potential as ligand molecules is still unexplored. Researchers Eissa and Zourob have presented a DNA aptasensor for the electrochemical detection of carbendazim, which is a fungicide applied in fields. The sensor was developed through aptamer coupling on gold electrodes by self-assembly of thiol and exploits the precise recognition of analytes by conformational changes in the aptamer structure [47]. Diverse approaches have been attempted for pesticide sensor establishment such as an impedimetric aptasensor developed for careful finding of acetamiprid and atrazine by micro-wires designed by platinum nanoparticles. The authors claim that this work was the first report of atrazine detection using an electrochemical aptasensor and lowered the detection limit to 1 pM [48]. Very recently, another report on electrochemical detection of chlorpyriphos used a reformative and discriminatory aptasensor based on a nanocomposite of copper oxide nanoflowers and single-walled carbon nanotubes. The LOD was improved to 70 pg/mL due to the synergistic effect of the nanocomposite [49]. The insecticide acetamiprid was detected using an electrochemical aptasensor on an unmodified gold electrode. This sensing strategy was created upon the target-induced release of MB from the dsDNA molded among aptamer and targeted complementary strand. The developed assay shows great sensitivity for acetamiprid and an LOD as low as 153 pM [50]. A nanostructure-based, highly sensitive electrochemical aptasensor was developed for the detection of chlorpyriphos. A novel composite mix of carbon black (CB) and graphene oxide@Fe_3_O_4_ was used and obtained a good linearity between 0.1 and 105 ng/mL with an LOD of 0.033 ng/mL. This sensor also showed good selectivity, reproducibility, and stability, and could be apply to screen chlorpyriphos in vegetable samples [51].

Even though aptamers offer striking advantages over antibody-based sensing techniques, they are still in the development phase as very limited numbers of aptamers are available for environmental contaminants. Another obstacle to constructing an aptasensor for environmental samples might be the poor immobilization strategies. There is enormous potential for developing novel electrochemical aptasensors for environmental monitoring. A summary of the electrochemical aptasensors applied for environmental safeguarding purposes is presented in Table 2.

## 5. Conclusions and Future Perceptions

The environment and food are exposed to a variety of hazardous lethal chemicals, which is damaging to the quality of human and animal life. Contaminants are continuously accumulating in the environment, which requires multipurpose and sophisticated sensing platforms for their detection. Thus, the development of routine monitoring systems with rapid and high-throughput analysis is needed. A combination of modern electrochemical tools with cutting-edge aptamer technology and microelectronics will provide powerful analytical methods for the efficient detection of these food and environmental contaminants. The requirements of an analytical system, such as sensitivity, selectivity, rapidity, cost-effectiveness, high-throughput analysis, and in-the-field analysis may be provided by the construction of effective electrochemical aptasensors using frontline and advanced technologies. The recent trends in the construction of such electrochemical aptasensors for real-time, on-site monitoring have effectively resolved the time constraints involved in conventional laboratory methods by providing high-throughput, multi-analyte, and portable biosensors. In a single foodstuff or environmental matrix, numerous co-contaminants may occur, and we anticipate that in the future a multi-analyte system may need to be developed for their simultaneous detection. The development of aptamer-based electrochemical biosensors for simultaneous detection of diverse target analytes might be challenging since the optimization of operational conditions and the immobilization of multiple aptamers on the sensor surface will be relatively complicated. This issue will be resolved by coupling realistically designed aptamers that can bind to different target analytes with the same affinity or by engineering new structure-switching aptamers for signal transduction upon binding with the target analyte. Furthermore, new aptamers for food-related and environmental contaminants and the integration of nanomaterials and lab-on-chip platforms for rapid, onsite monitoring of food and environmental samples are anticipated to be developed. The future will see a major increase in research dedicated to multi-analyte electrochemical nano-aptasensors for food and environmental safeguarding.

## Figures and Tables

**Table 1:** Selected electrochemical aptasensors applied for food safeguarding.

Electrochemical Aptasensors Applied for Food Safeguard				
Analyte	Matrix	Method details	Linear range and LOD	Reference
*Salmonella Typhimurium*	Pork	EIS based on AuNPs and GO	2.4–2.4 × 10^3^ CFU/mL, 3 CFU/mL	[17]
*Staphylococcus aureus*	Pig Skin	Potentiometry using SWCNT	800 CFU/mL	[18]
Lysozyme	wine	Aptamer–antibody diazonium coupling, DPV	5 fM–5 nM, 4.3 fM	[21]
Ochratoxin A	cocoa beans	Anti-OTA-aptamer on SPCE, EIS	0.15–2.5 ng/mL, 0.15 ng/mL	[22]
Aflatoxin M1	Milk	Hexaethyleneglycol modified aptamers on SPE, EIS	2–150 ng/L, 1.15 ng/mL	[23]
Aflatoxin B1	Alcoholic beverage	MB-tagged aptamer on SPCE, hexamethylenediamine and carbodiimide, DPV	0.05–6.0 ng/mL, 0.05 ng/mL	[6]
Kanamycin	Milk	Aptamer on SPCE, EIS	1.2–75 ng/mL, 0.11 ng/mL	[25]

**Table 2:** Selected electrochemical aptasensors applied for environmental safeguarding.

Electrochemical Aptasensors Applied for Environmental Safeguard				
Analyte	Matrix	Method details	Linear range and LOD	Reference
Hg^2+^	Drinking water	Aptamer, thymine-Hg^2+^-thymine nanoporous gold, DPV	0.01–5000 nM, 0.0036 nM	[29]
Hg^2+^	Environmental samples	dsDNA on Au electrode, Fe(CN)_6_^3−/4−^, DPV	5 zM–55 pM, 0.6 zM	[30]
Pb^2+^	Herbs	ferrocene-labeled thiolated aptamer, amperometry	5.0 × 10^−10^–1.0×10^–7^ M, 1.2 × 10^−10^ M	[31]
Chlorpyriphos	Vegetables and fruits	Aptamer on mesoporous carbon, chitosan and MWCNTs-CS, CV	1–10^5^ ng/mL, 0.33 ng/mL	[32]
